# Microbiologically Confirmed Primary Tubercular Breast Abscess in a Young Female: A Case Report

**DOI:** 10.1002/ccr3.70493

**Published:** 2025-05-13

**Authors:** Amanuel Zeleke, Tilahun Bizuayehu, Gashaw Solela, Sebhatleab Teju, Tesfamariam Gebreamlak, Abraham Adamu, Wondwossen Amogne

**Affiliations:** ^1^ Department of Internal Medicine Yekatit 12 Hospital Medical College Addis Ababa Ethiopia; ^2^ Infectious Diseases Unit, Department of Internal Medicine College of Health Sciences Addis Ababa Ethiopia; ^3^ Department of Internal Medicine College of Health Sciences, Addis Ababa University Addis Ababa Ethiopia; ^4^ Department of Internal Medicine Addis Hiwot General Hospital Addis Ababa Ethiopia; ^5^ Department of Oncology St. Paul's Hospital Millennium Medical College Addis Ababa Ethiopia

**Keywords:** breast abscess, case report, Ethiopia, tuberculosis, young female

## Abstract

Tuberculosis rarely affects the breast. This report presents a case of a microbiologically confirmed primary tubercular breast abscess in a young Ethiopian woman. A 26‐year‐old woman presented with left breast swelling and pain for 1 year. She was initially suspected to have a pyogenic abscess of the breast and was treated with unspecified oral antibiotics but had no clinical response. In addition, surgical drainage was done, followed by a 4‐week course of oral amoxicillin‐clavulanic acid. However, the swelling gradually increased in size and formed a sinus tract with a purulent discharge. Subsequently, breast tuberculosis was suspected, and a GeneXpert from the discharge was performed, which revealed rifampicin‐sensitive *Mycobacterium tuberculosis* . She was started on anti‐tuberculous medications and showed significant clinical response after 6 months of therapy. Breast tuberculosis should be considered earlier in patients with breast lesions in the absence of an alternative diagnosis and/or if the response to antibiotics is not as expected. In this case, histopathology from the breast lesion and cartridge‐based nucleic acid amplification test (GeneXpert) from the breast discharge were confirmatory for breast tuberculosis.

**Summary**

- Breast tuberculosis should be strongly considered as a differential diagnosis in patients presenting with breast lesions that do not respond to the recommended therapies, such as antibiotics, especially in high tuberculosis burden countries like Ethiopia.

## 1 Introduction

Tuberculosis (TB) is a chronic granulomatous infection caused by *Mycobacterium TB* (*MTB*) and a common disease in low‐ and middle‐income countries of the world. TB primarily affects the lungs but can also affect other organs (extra‐pulmonary), such as the lymph nodes, genitourinary system, bones and joints, gastrointestinal system, central nervous system, and spine [1]. The breast is an uncommon site for extra‐pulmonary tuberculosis, particularly as a primary presentation, even in regions where tuberculosis is endemic. It predominantly occurs in women of reproductive age [2].

Tuberculosis accounts for approximately 0.025%–0.1% of all surgically treated breast diseases, with a higher prevalence in developing countries [3]. The factors thought to be responsible for the higher incidence of the disease in the developing world include multiparity, lactation, trauma, past history of suppurative mastitis, and retroviral infections [4].

The clinical presentation of BTB is often insidious and non‐specific, manifesting as a breast abscess, a unilateral painless mass, or a sinus [5, 6]. Such presentations can mimic that of breast cancer, where the lesion could be characterized as being irregular, hard, and at times fixed to either skin or muscle or even the chest wall [7]. Clinically and radiologically, BTB can resemble a pyogenic breast abscess, fibroadenoma, or carcinoma [8, 9], the latter being the most concerning misdiagnosis because of the inherent consequences of inappropriate interventions such as surgery and/or chemotherapy.

Suspected breast tuberculosis (BTB) diagnosis hinges on microbiological and histopathologic findings. While microbiological culture of clinical samples is the gold standard, due to higher diagnostic value and phenotypic drug resistance testing, faster methods like acid‐fast bacilli (AFB) smear and polymerase chain reaction (PCR) based tests are usually preferred. Due to the paucibacillary nature of these specimens, test sensitivity is low, with AFB smear and culture detecting the bacilli in only 12% and 25% of cases, respectively. PCR's higher accuracy makes it essential for diagnosis in culture negative and/or smear‐negative cases [2, 4]. Histological findings on biopsy or fine‐needle aspiration cytology (FNAC) often suffice for diagnosis, especially in TB endemic regions, where FNAC achieves 73% sensitivity when signs of granulomas and necrosis are identified [10]. However, FNAC alone can lead to difficult differential diagnosis in cases of granulomatous mastitis and sarcoidosis, whose treatment (e.g., steroids) can lead to TB dissemination, highlighting the importance of microbiological testing [2].

This report illustrates a case of a PCR‐based rapid molecular test (GeneXpert) for confirming BTB encountered in a tertiary hospital in Ethiopia and highlights the salient features that may aid in diagnosis.

### 1.1 Case History and Physical Examination

A 26‐year‐old nulliparous woman presented with a 1‐year history of left breast painful swelling, which was slowly progressive in size. She was suspected to have a pyogenic breast abscess, and the patient reported that she received unspecified and various oral antibiotics at the nearby health facilities at different times for a year, but she had no clinical response. Progressively, she developed purulent discharge from the swelling and a sinus tract. An open surgical drainage was done, followed by a 4‐week course of oral amoxicillin‐clavulanic acid. Without any improvement, the abscess continued to drain pus, and she was finally referred to an oncology unit for evaluation and possible chemotherapy. She had no history of chronic cough, fever, night sweats, or weight loss. She did not report a history of other comorbid illnesses or a family history of breast cancer.

**FIGURE 1:**
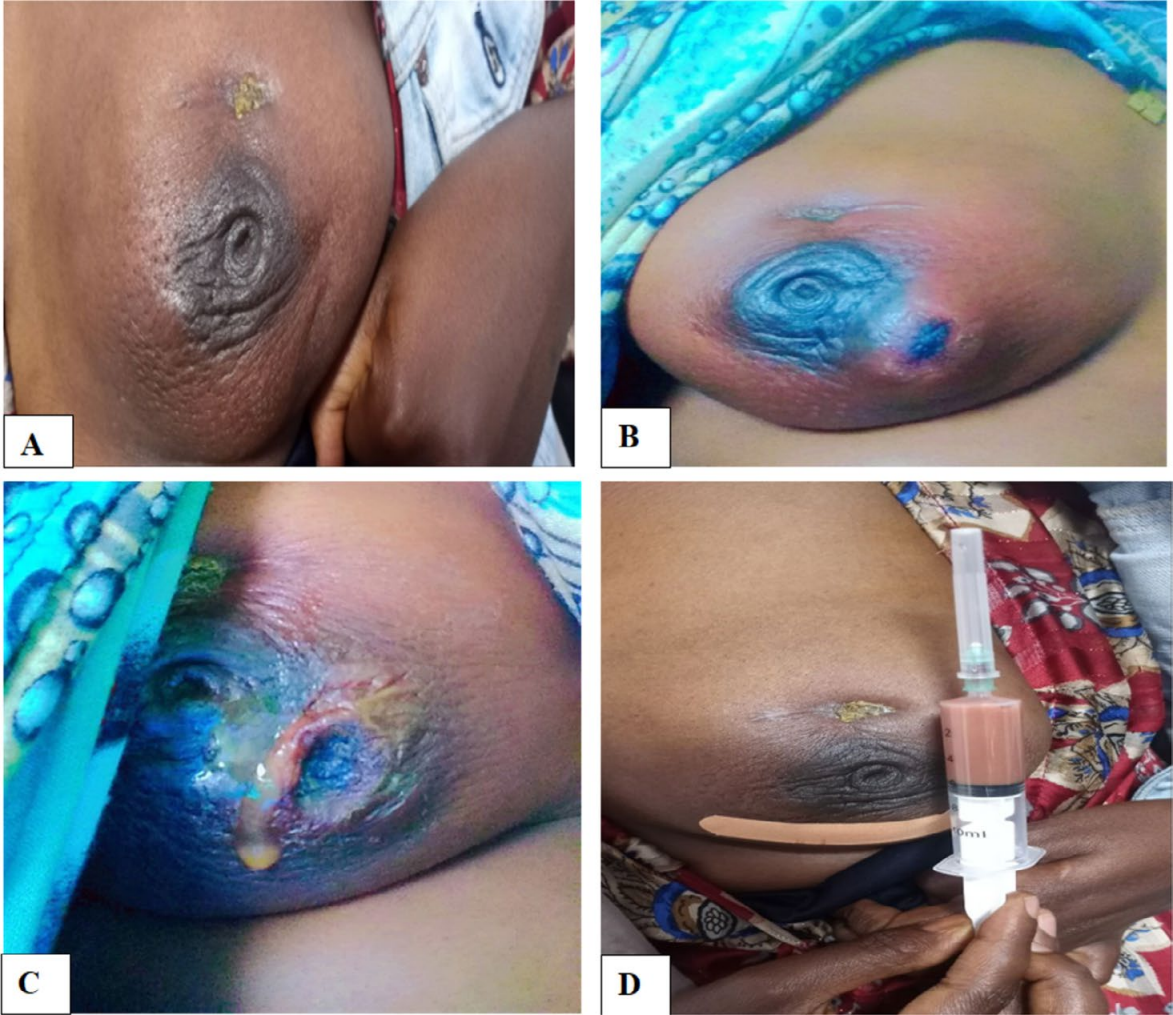
A 3‐cm painful, fluctuant mass in the upper outer quadrant of the left breast with a peau d'orange appearance and nipple retraction (A, B). There was a superficial ulcer over the mass with a pus‐draining tract (C). Aspiration of the mass using a syringe at bedside showed a thin, purulent collection (D).

The physical examination revealed a 3‐cm painful, fluctuant mass in the upper outer quadrant of the left breast with a peau d'orange appearance and nipple retraction (Figure 1A,B). There was a superficial ulcer over the mass with a pus‐draining tract (Figure 1C). Aspiration of the mass using a syringe at bedside showed a thin purulent collection (Figure 1D).

### 1.2 Differential Diagnosis, Investigations and Treatment

Important differential diagnoses in this case include idiopathic granulomatous mastitis (IGM), BTB, chronic pyogenic abscess, and inflammatory breast cancer. IGM is favored by the patient's age, chronic course of the disease, sinus formation, and poor response to antibiotics. Confirmation requires core biopsy showing non‐caseating granulomas and negative stains for infectious agents [11]. BTB is a key differential diagnosis, particularly in TB‐endemic regions. It often presents as a chronic breast abscess with discharging sinuses and may lack constitutional symptoms. In the present case, features supportive of BTB include the chronicity of the disease course, poor response to broad‐spectrum antibiotics, and the formation of sinuses. The diagnosis is confirmed by acid‐fast bacilli (AFB) staining, mycobacterial culture, or GeneXpert testing of pus or biopsy tissue [7, 8].

A chronic pyogenic breast abscess is still a possibility due to the ongoing purulent discharge; however, the lack of response to repeated antibiotic treatments makes it less likely unless resistant organisms are present. To confirm this, bacterial culture and sensitivity testing of the aspirated pus is needed to identify any atypical or resistant pathogens [12]. Although the patient's age and purulent discharge make inflammatory breast cancer less likely, its presentation with peau d'orange and nipple retraction warrants consideration. Despite being rare in young women, its aggressive nature and potential to mimic infection emphasize the need for histopathological evaluation to exclude malignancy [13].

Her hemoglobin level was 12 g/dL, white blood cell count of 10.5 × 10^3^/μL with a lymphocytosis of 49% and an erythrocyte sedimentation rate of 70 mm/h. With a high index of suspicion for BTB, GeneXpert was sent from the discharge, and it revealed a rifampicin‐susceptible *MTB*. Provider‐initiated counseling and testing for human immunodeficiency virus was nonreactive.

**FIGURE 2:**
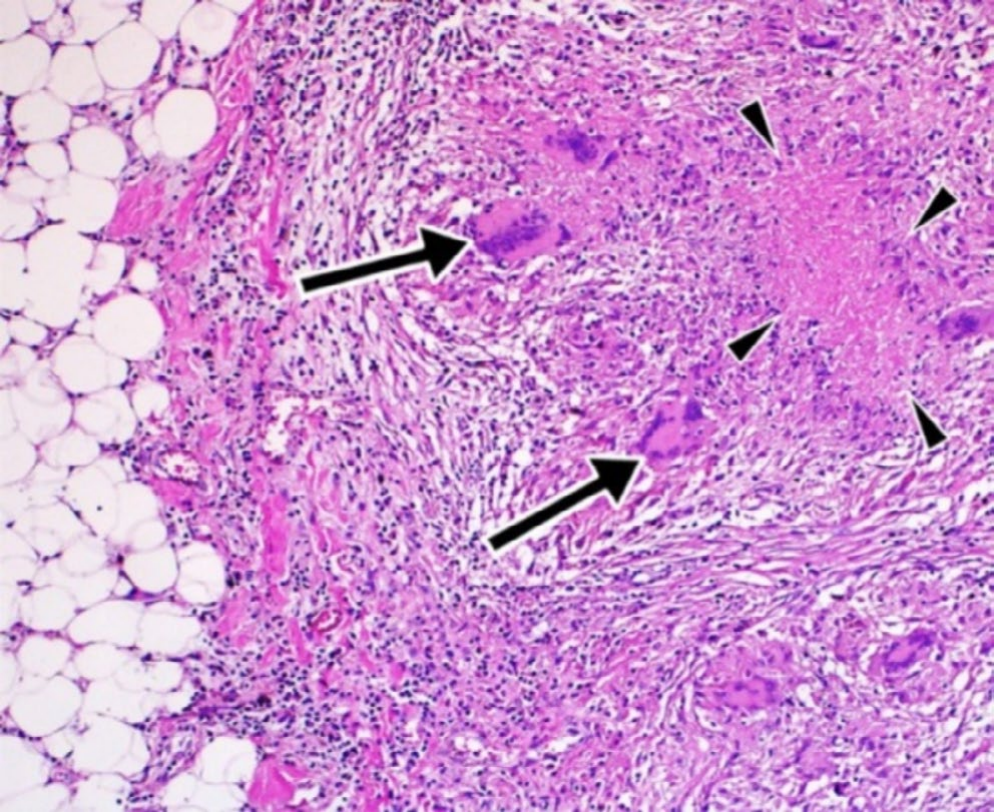
Histopathologic evaluation of the breast tissue revealing granulomatous inflammation, as evidenced by the long arrows indicating Langerhans‐type giant cells and the short arrows depicting caseous necrosis. The sample was stained with hematoxylin and eosin (H&E) and examined at 40× magnification.

Ultrasound of the left breast revealed a 4‐cm hypoechoic lesion with internal debris, thickened walls, surrounding fat stranding suggestive of abscess, and small‐sized left‐sided intramammary and axillary lymphadenopathies. Ultrasound‐guided core needle biopsy taken from the left breast abscess wall showed granulomatous mastitis described as cord‐like tissue fragments with dispersed epithelioid cell aggregates and a few Langerhans‐type giant cells. There were breast lobules surrounded by mixed inflammatory cell infiltrates (lymphoplasmacytic). No features of malignancy were seen (Figure 2). The chest X‐ray was normal.

### 1.3 Outcome and Follow‐Up

**FIGURE 3:**
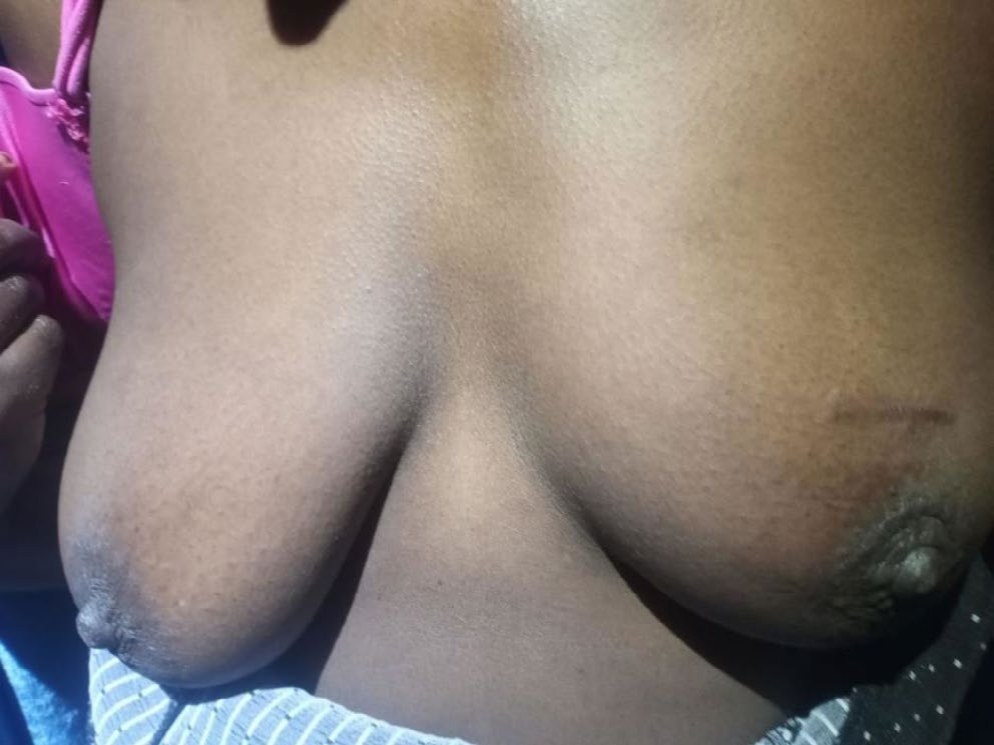
Healed left‐sided tubercular breast abscess after completion of anti‐TB treatment.

Six months after commencing anti‐TB therapy (rifampicin 150 mg, isoniazid 75 mg, pyrazinamide 400 mg, ethambutol 275 mg, 4 tablets daily), the lesion had healed completely (Figure 3), and the repeat breast ultrasound showed a remarkable improvement. Her ESR improved from the baseline. Additionally, she did not experience significant adverse effects from anti‐TB therapy, such as drug‐induced liver injury. She was followed for another 6 months after completing treatment, with no evidence of BTB recurrence.

## 2 Discussion

Tuberculosis was the leading infectious cause of death worldwide in 2023, with an estimated 10.6 million people globally and claimed approximately 1.25 million lives [14]. A systematic review and meta‐analysis of 20 studies conducted in Ethiopia between 2003 and 2021 reported a pooled prevalence of 39% for bacteriologically confirmed extrapulmonary tuberculosis based on the Xpert *MTB*/RIF assay. However, no cases of BTB were identified in this analysis [15]. This case report presents the diagnosis and management of an unusual type of extrapulmonary tuberculosis in a young female patient from Ethiopia.

It is generally believed that the infection of the breast is usually secondary to a primary site elsewhere in the body, which may or may not be clinically apparent; however, breast TB may be the primary site when no demonstrable tuberculous focus exists elsewhere [4]. BTB is classified into three forms: the nodular form (painless, slowly growing mass), the disseminated form (multiple lesions with sinus tracts), and the abscess form (fluctuating mass, commonly seen in younger women) [7, 16].

BTB remains an exceedingly rare presentation, even in regions with a high prevalence of tuberculosis. The prevalence of the disease ranges from less than 1% in the industrialized world to as high as 4% in the Indian subcontinent [5]. Lactating women in the reproductive age group are the most commonly affected, although case reports of males with BTB have been reported [2, 6]. The reason why it occurs during the period of childbearing age is hypothesized to be due to increased vasculature and a higher risk of repetitive trauma and infection [2, 4].

Despite the commonly identified risk factors for BTB, such as multiparity, lactation, a history of suppurative mastitis, and retroviral infections [2], our patient did not have any of these. She also had no known chronic illnesses, such as diabetes. This suggests that tuberculosis can affect any part of the body, regardless of the presence or absence of known risk factors.

BTB often presents as a unilateral, painful, and poorly defined breast lump persisting for months [8]. A review of 33 granulomatous BTB cases found that over one‐third of patients had multiple clinic visits before being diagnosed, with an average delay of 3.7 months [17], similar to our case. Physical examination may reveal sinuses, fistulous tracts, superficial ulcers, axillary lymphadenopathy, and even multiple masses, which are usually found in the central and upper outer parts of the breast, as observed in our case [8].

The diagnosis of extrapulmonary TB is challenging, requiring a targeted imaging approach accompanied by appropriate histopathologic examination and microbiologic testing [18]. Breast ultrasound is crucial in diagnosing BTB, as it can identify solitary or multiple masses, inflammatory changes, sinus tracts, or lymphadenopathy. Additionally, it serves as a guide for diagnostic and therapeutic aspirations, drainages, and biopsies for histopathological and bacteriological confirmation [2]. In our case, breast ultrasound played a great role in revealing a left‐sided breast abscess along with ipsilateral intramammary and axillary lymphadenopathies, though the major caveat was its inability to identify the underlying cause.

Unlike pulmonary TB, extrapulmonary TB sites are often biopsied due to their typically pauci‐bacillary nature and frequent negative cultures, and the typical histopathological features of tuberculous granulomas provide strong evidence for a TB diagnosis [18]. The presence of a granulomatous lesion on biopsy with Langerhans‐type giant cells from the breast, as demonstrated in our patient and other case reports [8, 19], may be a suggestive feature even if it is not a confirmatory result.

The gold standard for the diagnosis of BTB is the detection of *MTB* by AFB staining, GeneXpert testing, or the isolation of the organism from the lesion on culture [2]. GeneXpert testing confirmed the diagnosis of BTB in a significant number of patients in a review of case reports and a retrospective case series, particularly in those presenting with breast abscesses [8, 20]. Similarly, the final diagnosis of BTB in our patient was confirmed using GeneXpert testing, highlighting its essential role in diagnosing BTB.

One major diagnostic challenge in BTB is its diverse clinical presentations and its masquerading as breast carcinoma, increasing the chance for misdiagnosis and unnecessary interventions [8, 9]. Our patient showed signs typically associated with breast carcinoma, such as nipple retraction and peau d'orange changes [13], which were also observed in other cases of BTB [8, 9]. BTB should be considered in regions with high TB prevalence, even if clinical features may mimic breast cancer, with biopsy and microbiological evaluation being crucial for accurate diagnosis.

BTB is effectively treated with anti‐TB therapy, and surgery is rarely needed [4]. A case series of 12 patients with BTB showed that all were treated with anti‐TB therapy, and only one patient presented with a recurrent breast abscess [21]. In another study of 17 cases, 64.7% of patients achieved full recovery by the end of treatment, 23.5% experienced recurrence, and most (76.5%) were treated for 9 months [9]. In our patient, anti‐TB therapy was administered for 6 months, leading to significant improvement, and there was no BTB recurrence during subsequent follow‐up.

## 3 Conclusions

BTB should be considered earlier in patients with breast lesions in the absence of an alternative diagnosis and/or if the response to antibiotics is not as expected. Histopathology from the breast lesion and GeneXpert from the breast discharge can be confirmatory for BTB, as established by this report.

## Author Contributions

**Amanuel Zeleke:** conceptualization, data curation, validation, writing – original draft, writing – review and editing. **Tilahun Bizuayehu:** conceptualization, data curation, validation, writing – original draft, writing – review and editing. **Gashaw Solela:** data curation, writing – review and editing. **Sebhatleab Teju:** conceptualization, data curation, validation, writing – original draft, writing – review and editing. **Tesfamariam Gebreamlak:** data curation, writing – review and editing. **Abraham Adamu:** data curation, writing – review and editing. **Wondwossen Amogne:** data curation, writing – review and editing.

## Ethics Statement

Ethical clearance was obtained from the Institutional Review Board (IRB) of Yekatit 12 Hospital Medical College. Consent to participate is not applicable in this case report.

## Consent

Written informed consent was obtained from the patient for publication of this case report and any accompanying images. A copy of the written consent is available for review by the Editor‐in‐Chief of this journal.

## Conflicts of Interest

The authors declare no conflicts of interest.

## Data Availability

Data supporting this case report will be available with the corresponding author upon reasonable request.
